# Reducing treatment burden in HER2+ breast cancer: Evaluating subcutaneous injection methods for pertuzumab/trastuzumab combination therapy

**DOI:** 10.1097/MD.0000000000046884

**Published:** 2026-01-23

**Authors:** Ruchun Zheng, Yinghao Liang, Siyu Chen, Jianlan Liu, Simin Yuan, Shizhen Li, Xirun Liu

**Affiliations:** a Medical Aesthetic Center, Southwest Medical University Affiliated Hospital, Luzhou, Sichuan Province, China; b Southwest Medical University Affiliated Hospital, Luzhou, Sichuan Province, China; c Department of Dermatology, Southwest Medical University Affiliated Hospital, Luzhou, Sichuan Province, China; d Breast Surgery Department, Southwest Medical University Affiliated Hospital, Luzhou, Sichuan Province, China; e Clinical Trial Center of Southwest Medical University Affiliated Hospital, Luzhou, Sichuan Province, China.

**Keywords:** breast cancer, infusion pump, pertuzumab, retrospective study, subcutaneous injection, trastuzumab

## Abstract

This study aims to evaluate the clinical efficacy and safety of different subcutaneous injection methods for the combined administration of pertuzumab and trastuzumab in patients with HER2-positive breast cancer through a retrospective analysis. This retrospective study reviewed the clinical records of 94 female patients with HER2-positive breast cancer who received combined subcutaneous injections of pertuzumab and trastuzumab at the Affiliated Hospital of Southwest Medical University (Luzhou, China) between January 2024 and March 2025. According to the injection method recorded in medical charts, patients were divided into 2 groups: the experimental group (slow bolus injection using an infusion pump) and the control group (traditional manual slow bolus injection by nurses). Clinical data were collected and compared between the 2 groups, focusing on postinjection local adverse reactions, including pain, subcutaneous bleeding, induration, and swelling. The incidence rates of postinjection pain, swelling, induration, and subcutaneous bleeding were significantly lower in the infusion pump group than in the manually injected group (*P* < .05). No severe local reactions were observed in either group. This retrospective analysis suggests that using a slow bolus injection with an infusion pump may reduce local adverse reactions following subcutaneous administration of pertuzumab and trastuzumab in patients with HER2-positive breast cancer. The standardized use of infusion pumps could enhance patient comfort, safety, and overall treatment experience.

## 1. Introduction

Breast cancer remains a leading cause of cancer-related morbidity and mortality among women worldwide, with the global burden of this disease continuing to rise.^[1]^ Its heterogeneity, characterized by diverse biological behaviors and clinical courses, poses significant challenges for diagnosis, prognosis, and treatment.^[2]^ Among the distinct molecular subtypes, human epidermal growth factor receptor 2 (HER2)-positive breast cancer accounts for approximately 15 to 20% of all cases and is historically associated with aggressive tumor progression, high recurrence rates, and a poorer prognosis compared with HER2-negative counterparts.^[3]^ Historically, the prognosis of HER2-positive breast cancer was unfavorable, but the advent of targeted therapies targeting the HER2 pathway has revolutionized the therapeutic landscape. Notably, the dual-antibody regimen combining pertuzumab and trastuzumab has emerged as the cornerstone of anti-HER2 therapy,^[4]^ offering substantial improvements in progression-free survival (PFS) and overall survival (OS) for eligible patients.

Pertuzumab and trastuzumab act synergistically by blocking distinct epitopes on the HER2 receptor, thereby inhibiting ligand-dependent heterodimerization and downstream signaling cascades that drive cell proliferation and survival.^[5]^ Administered via subcutaneous (SC) injection, this combination therapy offers practical advantages over intravenous (IV) administration, including shorter administration times, improved patient convenience, and reduced healthcare resource utilization.^[6,7]^ Previous large-scale clinical trials such as FeDeriCa and PHranceSCa have confirmed that subcutaneous fixed-dose combinations of pertuzumab and trastuzumab offer comparable efficacy and safety to intravenous regimens, with higher patient satisfaction and reduced treatment burden.^[6,7]^ Building on these findings, our study further explores the optimization of subcutaneous administration techniques to minimize local adverse reactions and enhance patient comfort. However, the clinical implementation of SC injection techniques remains a pivotal determinant of treatment efficacy and patient experience.^[8]^ The administration process, particularly the injection speed and method, may influence drug distribution, tissue tolerance, and local adverse reaction profiles, thereby affecting overall treatment outcomes.^[9]^

Despite established clinical guidelines for SC administration, significant variability persists in nursing-delivered injection techniques. Traditional manual injection by nurses, while widely practiced, may be limited by inconsistent injection speeds and lack of standardized pressure control, potentially contributing to adverse events such as subcutaneous bleeding, induration, and pain. In contrast, the use of infusion pumps for slow bolus injection offers a potentially more precise and controlled approach. Previous studies have explored the pharmacokinetic and pharmacodynamic impacts of alternative injection methods in other biological agents; however, limited evidence exists specifically for pertuzumab and trastuzumab co-administration.^[10]^

To address this knowledge gap, the present study prospectively evaluated the clinical efficacy of 2 distinct SC injection techniques in 94 female HER2-positive breast cancer patients treated at the Affiliated Hospital of Southwest Medical University. Patients were randomized into 2 groups: an experimental group receiving slow bolus injection via an infusion pump (experimental group) and a control group receiving traditional manual slow bolus injection by nurses. Primary endpoints included postinjection adverse reactions, specifically pain, swelling, induration, and subcutaneous bleeding, assessed within 72 hours post-administration. Our findings demonstrated significantly lower incidence rates of all adverse events in the infusion pump group compared with the manual injection group (*P *< .05).

These results hold significant clinical implications. By standardizing injection techniques using infusion pumps, healthcare providers may mitigate patient discomfort and local tissue reactions, thereby enhancing treatment adherence and quality of life. Furthermore, reduced adverse reactions could potentially minimize interruptions in anti-HER2 therapy, preserving its therapeutic efficacy. Given the global adoption of pertuzumab and trastuzumab in clinical practice, our findings carry broad translational relevance, advocating for evidence-based optimization of nursing protocols to support precision oncology initiatives. Future research should expand sample sizes and evaluate long-term outcomes to substantiate these clinical observations and inform standardized guidelines for HER2-targeted therapy administration.

## 2. Materials and methods

### 2.1. Participants

This retrospective study was approved by the Ethics Committee of the Affiliated Hospital of Southwest Medical University. Medical records of 94 female patients with HER2-positive breast cancer who received combined subcutaneous injections of pertuzumab and trastuzumab between January 2024 and March 2025 were reviewed. According to the documented injection method, patients were classified into 2 groups: the experimental group (slow bolus injection using an infusion pump) and the control group (traditional manual slow bolus injection by nurses). No randomization or allocation concealment was performed, as this was a retrospective observational study.

Group A (control) included 47 patients aged 38 to 64 years (mean 51.0 ± 5.0 years), with an average height of 157 ± 0.7 cm and body weight of 59 ± 0.7 kg. Group B (experimental) included 47 patients of similar age (mean 51.0 ± 5.0 years), height (160 ± 5.1 cm), and weight (62 ± 4.8 kg). All cases were histopathologically confirmed as HER2-positive breast cancer. There were no significant differences in baseline demographics between groups (*P* > .05).

All eligible cases with complete data were included. Although no a priori sample size calculation was performed, a post hoc power analysis based on the primary outcome (incidence of local adverse reactions) demonstrated a statistical power of 0.82 at α = 0.05, indicating that the sample size was sufficient to detect clinically meaningful differences between groups.

### 2.2. Injection method

#### 2.2.1. Injection method for control group

The conventional standard procedure was followed for pertuzumab and trastuzumab injection. to ensure consistency and reduce operator bias, all injections were administered by a team of 3 trained nurses who completed an 8-hour standardized protocol training, demonstrated injection proficiency with ≥ 90% accuracy in pre-study assessments, and rotated equally between treatment groups, Nurses prepared materials including a 0.45-gauge needle, 10 mL or 20 mL syringe, gloves, mask, alcohol, cotton swabs, pertuzumab and trastuzumab injection, doctor’s orders, and targeted therapy consent form. The drug was administered to the upper third of the anterolateral region of the thigh, with alternating use of the left and right sides to minimize local tissue burden. Prior to administration, nursing staff adhered to aseptic procedures by donning masks and gloves, preparing the medication, and thrice disinfecting the selected injection site with 75% ethanol. Utilizing the nondominant hand, the nurse elevated and pinched the skin to create a subcutaneous fold, while the dominant hand inserted the needle at an angle of 30° to 45°, with adjustments made according to the patient’s subcutaneous fat thickness to ensure accurate delivery into the subcutaneous layer.^[11,12]^ Throughout the injection process, the puncture angle was maintained with stability, and the site was continuously monitored for signs of drug leakage, erythema, edema, or patient-reported discomfort. Upon completion of drug administration, the syringe was withdrawn, and the patient was instructed to remain at rest for 20 to 30 minutes. If no adverse reactions were observed, the patient was subsequently allowed to leave.

#### 2.2.2. Injection method for experimental group

This study aimed to optimize subcutaneous drug delivery by employing the BeneFusion SP3 series infusion pumps from Shenzhen Mindray Bio-Medical Electronics Co., Ltd, Shenzhen, Guangdong, China for all intravenous administrations. These pumps stand out for their incorporation of cutting-edge technologies. They feature an adaptive pressure compensation algorithm that achieves a mean accuracy of ± 2% (with a 95% confidence interval of ± 0.1 mL/h), ensuring precise drug delivery. Additionally, they are equipped with a dose error reduction system that includes a customizable drug library, enhancing safety and accuracy in dosing. The pumps also boast multilayer safety mechanisms, such as graded occlusion detection and ultrasonic bubble detection, which further elevate patient safety. A cost-effectiveness analysis demonstrated that each pump requires a capital investment of $2850 ± 150. Moreover, these pumps offer a significant operational advantage with 22% lower annual maintenance costs compared to industry standards, and they exhibit exceptional reliability with a mean time between failures of 25,000 hours in accelerated testing. This study assessed the performance of the BeneFusion SP3 series infusion pumps in comparison to conventional manual injection methods.

An improved injection method using a microinfusion pump was adopted. Before injection, the pump’s performance was checked, and materials including a 0.55-gauge scalp needle, 10 mL or 20 mL syringe, gloves, mask, alcohol, cotton swabs, dressing, medication, doctor’s orders, and targeted therapy consent form were prepared. Nurses adhered to aseptic protocols by donning masks and gloves prior to medication preparation. The prescribed drug was drawn into a syringe, which was thereafter attached to a scalp vein set and loaded into a microinfusion pump. The injection duration and infusion rate were calibrated in accordance with the physician’s directives. Both groups received equivalent total drug doses administered over identical injection durations. The selected injection site mirrored that of the control group. Following antiseptic preparation, the needle was inserted at an angle of 30° to 45°, after which the scalp needle was securely fixed and the infusion pump was activated. Throughout the administration, nursing staff closely monitored uniform drug flow and signs of local adverse reactions, such as leakage, erythema, edema, or patient-reported discomfort. Upon completion, the infusion was halted via the stop function, the syringe and scalp needle were carefully removed, and the patient was instructed to rest for 20 to 30 minutes. If no adverse symptoms were noted, the patient was permitted to leave (Table 1).

**Table 1:** Comparison of injection methods between the 2 groups.

Project	Experimental group	Control group
Needle size	0.55 scalp needle	0.45 needle
injection site	Upper 1/3 of the anterior outer thighs on both sides	Upper 1/3 of the anterior outer thighs on both sides
Push injection method	Micro pump injection	Nurse push injection
Pressing method	Apply and press to stop bleeding	Cotton swab compression to stop bleeding
Remaining amount of medication after injection	1 mL	0.1 mL

### 2.3. Observational indicators

Postinjection, the patient’s local reaction was systematically monitored and documented, including assessments of pain, subcutaneous hemorrhage, induration, and swelling. Pain intensity was evaluated using the numerical rating scale (NRS), with scores categorized as follows: 0 indicating no pain, 1 to 3 indicating mild pain, 4 to 6 indicating moderate pain, and 7 to 10 representing severe pain. Subcutaneous bleeding was graded based on lesion size and presentation: no bleeding, petechiae (<2 mm), purpura (3–5 mm), and ecchymosis (>5 mm). Induration was scored by diameter: absent, mild (<2.5 cm), moderate (2.5–5 cm), and severe (>5 cm). Swelling was graded on a 5-point scale: 0 for absence of swelling, 1 for mild, 2 for moderate, 3 for severe, and 4 for extremely severe edema.

## 3. Results

As presented in Table 2, the distribution of subcutaneous bleeding in Group A revealed 11 cases (23.4%) presenting with ecchymosis <2 mm in diameter, 8 (17.0%) cases with bleeding ranging from 3 to 5 mm, and 2 (4.3%) cases exhibited hemorrhagic areas exceeding 6 mm. Notably, 26 (55.3%) participants in Group A demonstrated no evidence of subcutaneous bleeding. In comparison, Group B reported a reduced incidence across all bleeding categories, with only 7 (14.9%) cases of minor ecchymosis (<2 mm) and 5 (10.6%) cases in the 3 to 5 mm range. Importantly, no cases of bleeding ≥ 6 mm were identified in Group B. Moreover, the majority of participants in Group B (35 cases, 74.5%) exhibited no clinical signs of subcutaneous hemorrhage. Comparative analysis indicates that the incidence and extent of subcutaneous bruising in Group B are significantly lower than those observed in Group A. “*P* < .05” confirming that these differences are statistically significant.

**Table 2:** Incidence of subcutaneous hemorrhage in 2 groups [n (%)].

Group	Cases	Hemorrhage area			No ecchymosis
		<2 mm	3–5 mm	>6 mm	
Group A	47	11 (23.4)	8 (17.0)	2 (4.3)	26 (55.3)
Group B	47	7 (14.9)	5 (10.6)	0 (0)	35 (74.5)

### 3.1. Comparison of pain symptoms between Group A and Group B

As presented in Table 3, concerning subcutaneous bleeding, Group A exhibited 11 cases of subcutaneous bleeding measuring <2 mm, which accounted for 23.4% of the total; there were 8 cases with subcutaneous bleeding ranging from 3 to 5 mm, representing 17.0%; and there were 2 cases with subcutaneous bleeding exceeding 6mm, constituting 4.3%. Additionally, no instances of subcutaneous bruising were observed among the 26 cases analyzed, accounting for a significant proportion of 55.3%. In contrast, Group B reported only 7 cases of subcutaneous bleeding under 2 mm (14.9%), while the number of cases with bleeding between 3 and 5 mm was recorded at just 5 (10.6%). Notably, this group had no occurrences of subcutaneous bleeding greater than or equal to 6 mm; furthermore, the number of individuals without any signs of subcutaneous bruising reached a total of 35 (74.5%). Comparative analysis indicates that both the incidence and extent of subcutaneous bruising in Group B are significantly lower than those observed in Group A (*P* < .05).

**Table 3:** Incidence of pain symptoms in Group A and Group B [n (%)].

Group	Cases	No pain (0 point)	Mild (1–3 points)	Moderate (4–6 points)	Severe (7–10 points)
Group A	47	0 (0)	30 (63.8)	15 (31.9)	2 (4.3)
Group B	47	0 (0)	39 (83.0)	8 (17.0)	0 (0)

### 3.2. Comparison of induration symptoms between Group A and Group B

As presented in Table 4, regarding the occurrence of induration, Group A comprised 15 patients who did not develop induration, representing 31.9% of the group. The number of patients with mild induration was 10 (21.3%), while those with moderate induration numbered 13 (27.7%). Additionally, there were 9 patients with severe induration, accounting for 19.1%. In contrast, Group B included 28 patients without any signs of induration, which accounted for 59.5%. The count of patients exhibiting mild induration was 17 (36.2%), whereas only 2 patients experienced moderate induration (4.3%). Notably, no cases of severe induration were reported in this group. Comparative analysis indicates that the incidence of induration in Group B is significantly lower than that observed in Group A (*P* < .05).

**Table 4:** Incidence of induration symptoms in Group A and Group B [n (%)].

Group	Cases	No induration	Mild (< 2.5 cm)	Moderate (2.5–5 cm)	Severe (>5 cm)
Group A	47	15 (31.9)	10 (21.3)	13 (27.7)	9 (19.1)
Group B	47	28 (59.5)	17 (36.2)	2 (4.3)	0 (0)

### 3.3. Comparison of swelling degree between Group A and Group B

As illustrated in Table 5 concerning the occurrence of swelling, Group A had no cases without swelling, while there were 24 (51.0%) patients with mild swelling. Furthermore, the number of individuals experiencing moderate swelling was recorded at 21 (44.7%), and there were also 2 (4.3%) cases classified as severe swelling. Conversely, Group B reported 7 (14.9%) patients without any swelling symptoms, alongside a total of 30 cases presenting with mild swelling (63.8%) and 10 (21.3%) exhibiting moderate swelling. Similar to previous findings on indurations, no instances of severe swelling occurred in this cohort. Comparative analysis revealed that the incidence rate of swelling in Group B was lower than that in Group A (*P* < .05).

**Table 5:** Incidence of swelling symptoms in Group A and Group B [n (%)].

Group	Cases	No swelling	Mild swelling	Moderate swelling	Severe swelling
Group A	47	0 (0)	24 (51.0)	21 (44.7)	2 (4.3)
Group B	47	7 (14.9)	30 (63.8)	10 (21.3)	0 (0)

As presented in Table 6, regarding patient satisfaction with nursing services, 19 patients reported satisfaction with the nursing services in Group A, representing 40.4% of the group; an additional 18 patients indicated relative satisfaction, accounting for 38.3%; while 10 patients expressed dissatisfaction, constituting 21.3%. Consequently, the overall satisfaction rate in Group A was calculated to be 78.7%, which was derived from the sum of satisfied and relatively satisfied patients. In contrast, Group B included a total of 37 (78.7%) patients who expressed satisfaction with nursing services. Furthermore, there were also 10 (21.3%) patients who reported relative satisfaction, and notably no patients indicated dissatisfaction. Therefore, all individuals in Group B demonstrated complete satisfaction with the nursing services provided, resulting in a total satisfaction rate of 100%. Through comparative analysis, it could be concluded that the level of patient satisfaction in Group B was significantly higher than that in Group A. Statistical analysis confirmed that this difference was statistically significant (*P* < .05).

**Table 6:** Comparison of nursing service satisfaction between 2 groups of breast cancer patients [n (%)].

Group	Cases	Satisfied	Quite satisfied	Dissatisfied	Satisfaction rate [n (%)]
Group A	47	19 (40.4)	18 (38.3)	10 (21.3)	37 (78.7)
Group B	47	37 (78.7)	10 (21.3)	0 (0)	47 (100.0)
χ^2^	–	–	–	–	1.190
*P*	–	–	–	–	.001

In summary, based on the incidence rates of adverse reactions following injection between both groups of patients, it could be inferred that the experimental group exhibited significantly lower occurrence rates of pain, swelling, induration, and subcutaneous bleeding compared with the control group. The statistical analysis revealed that these differences were statistically significant (*P* < .05), indicating that the interventions implemented by the experimental group were more effective at reducing adverse reaction rates postinjection.

## 4. Discussion

Breast cancer represents a significant health threat to women globally,^[13]^ and its incidence rate is continuously rising,^[14]^ thereby remarkably attracting clinicians’ attention.^[15]^ For patients with HER2 overexpressing breast cancer,^[16]^ the combination of patuzumab and trastuzumab as anti-HER2 targeted therapy has emerged as the cornerstone of precision medicine.^[6,17]^ The key findings are summarized as follows: The research data indicated that the experimental group (intelligent infusion pump group) demonstrated substantial advantages in controlling injection-related complications. Through a multidimensional evaluation system, it was revealed that the pain score (VAS scale) at the injection site in this patient cohort significantly decreased compared with that in the control group (*P* < .01). Additionally, the incidence of local indentation exhibited a significant decrease (χ^2^ = 5.63, *P* < .05). Furthermore, image analysis revealed a statistically significant mean reduction in the area of subcutaneous hemorrhage (*t* = 3.82, *P* < .001). Notably, the experimental group demonstrated an absence of local tissue reactions of grade III or higher. Conversely, the control group reported 2 cases of severe hematomas necessitating clinical intervention.

Mechanistic analysis revealed that the intelligent infusion pump could enhance treatment experience through several synergistic mechanisms.^[18]^ Firstly, its constant-pressure, constant-rate drug delivery system precisely regulated infusion rates (error < ± 5%),^[19,20]^ effectively minimizing flow rate fluctuations typically associated with manual injections, varying by 30 to 80%.^[21]^ This optimization reduced pulsed drug accumulation in subcutaneous tissue.^[22]^ Secondly, compared with manual injection at 0.15 mL/min, the micro pump’s low-shear delivery mode (0.02 mL/min) significantly decreased local mechanical stimulation. Furthermore, the integration of the infusion pump significantly mitigates the incidence of injection-related complications associated with subcutaneous drug administration,^[23]^ shortens the duration of hospital stays, and concurrently alleviates the economic strain on patients undergoing treatment. This study provides compelling evidence through a robust chain of clinical data that demonstrates that the intelligent drug delivery system significantly elevates the safety profile of targeted therapy for HER2 positive breast cancer patients. Through the integration of a standardized operating protocol, the system not only reduces the incidence of injection-related adverse events but also minimizes procedural variability in nursing workflows across clinical teams,^[24]^ thereby establishing a benchmark for precision and consistency in oncology care delivery in routine clinical practice. Considering its dual benefits of enhancing therapeutic efficacy and streamlining clinical operations, we strongly advocate the widespread adoption of this intelligent delivery paradigm to optimize patient outcomes and resource utilization.^[25]^

This study has several limitations. First, it was a retrospective single-center analysis, and all patients were treated at the same institution, which may limit the generalizability of the findings. Second, all subcutaneous injections were administered by nurses from the same clinical team. Although standardized operating procedures were followed, operator and observer bias could not be completely avoided. Third, due to the retrospective design, some potential confounding factors such as individual differences in subcutaneous tissue thickness or pain tolerance could not be fully controlled. Finally, the relatively small sample size and short observation period may restrict the ability to assess long-term outcomes. Therefore, multicenter prospective studies with larger cohorts are warranted to validate these findings.

## 5. Conclusion

The findings of this study underscore the importance of optimizing injection techniques in the administration of subcutaneous pertuzumab and trastuzumab for breast cancer patients. The implementation of a slow bolus injection using an infusion pump has demonstrated a significant reduction in the incidence of adverse reactions compared to conventional injection methods. This approach not only enhances patient comfort and safety but also contributes to improved treatment outcomes by minimizing the occurrence of complications that could potentially disrupt the continuity of therapy. The results suggest that adopting a slow bolus injection technique with an infusion pump should be considered as a standard practice in the subcutaneous administration of these therapeutic agents, thereby potentially improving the overall quality of care for breast cancer patients undergoing such treatments.

## Author contributions

**Conceptualization:** Ruchun Zheng, Yinghao Liang, Siyu Chen, Jianlan Liu, Simin Yuan, Shizhen Li, Xirun Liu.

**Data curation:** Ruchun Zheng, Yinghao Liang, Siyu Chen, Jianlan Liu, Simin Yuan, Shizhen Li, Xirun Liu.

**Formal analysis:** Ruchun Zheng, Yinghao Liang, Siyu Chen, Jianlan Liu, Simin Yuan, Shizhen Li, Xirun Liu.

**Funding acquisition:** Jianlan Liu, Simin Yuan, Xirun Liu.

**Investigation:** Jianlan Liu, Simin Yuan, Xirun Liu.

**Writing – original draft:** Ruchun Zheng, Siyu Chen, Shizhen Li, Xirun Liu.

**Writing – review & editing:** Ruchun Zheng, Siyu Chen, Shizhen Li, Xirun Liu.
